# Anisotropic Sensing Performance in a High-Sensitivity Surface Plasmon Resonance Sensor Based on Few-Layer Black Phosphorus

**DOI:** 10.3390/s24123851

**Published:** 2024-06-14

**Authors:** Qifeng Zhu, Yanting Shen, Zhuo Chen, Binghuang Chen, Enwen Dai, Weiqing Pan

**Affiliations:** Department of Physics, Zhejiang University of Science and Technology, Hangzhou 310023, China

**Keywords:** few-layer black phosphorus, surface plasmon resonance, stacking sequences, sensors

## Abstract

Few-layer black phosphorus (FLBP) is a highly promising material for high sensitivity label-free surface plasmon resonance (SPR) sensors due to its exceptional electrical, optical, and mechanical properties. FLBP exhibits inherent anisotropy with different refractive indices along its two main crystal orientations, the zigzag and armchair axes. However, this anisotropic property is often overlooked in FLBP-based sensors. In this study, we conducted a comprehensive investigation of the SPR reflectivity and phase in a BK7-Ag-FLBP structure to understand the influence of the stacking sequence and the number of FLBP layers on the sensing performance. Clear resonant angle shifts caused by different stacking sequences of FLBP could be observed both theoretically and experimentally. In the theoretical study, the highest reflective and phase sensitivities were achieved with a 12-layer black phosphorus (BP) structure. The reflectivity sensitivity reached 287.9°/refractive index units (RIU) with the zz stacking 12-layer BP film exhibiting a sensitivity 76°/RIU higher than the ac stacking structure. Similarly, the phase sensitivity reached 1162°/RIU with the zz stacking 12-layer BP structure showing a sensitivity 276.9°/RIU higher than the ac stacking structure. The electric field distribution of the 12-layer BP structure with four different stacking sequences has also been analyzed. In the experiment study, the well-known Attenuated Total Reflection (ATR) θ−2θ SPR setup is utilized to detect the reflectivity and phase of BK7-Ag-FLBP structures. The FLBP samples with the same thickness but different stacking sequences show significant resonant angle shift (0.275°) and maximum phase difference variation (34.6°). The FLBP sample thickness and crystal orientations have been demonstrated using the angular-resolved polarized Raman spectroscopy (ARPRS). These theoretical and experimental results provide strong evidence that the stacking sequences of FLBP have a significant impact on the sensing performance of SPR sensors. By harnessing the anisotropic properties of materials like FLBP, novel structures of anisotropic-2D material-based SPR sensors could open up exciting possibilities for innovative applications.

## 1. Introduction

Surface plasmon resonance (SPR) is a phenomenon that occurs when light interacts with a metal–dielectric interface, typically involving noble metals such as gold or silver. It is based on the collective oscillation of free electrons, known as surface plasmons (SPs), at the metal–dielectric interface [1]. The SPR condition is extremely sensitive to the refractive indices of both the metal and dielectric media, and any change in either causes the resonance parameter to change. By manipulating the initial momentum of the incoming p-polarized light, it is possible to excite an SPP. One way to accomplish this is by transmitting the light through high refractive index prisms within an attenuated total internal reflection (ATR) probe, such as the Kretschmann configuration, which was first demonstrated experimentally in 1968 [1,2]. Since then, SPR sensors have emerged as powerful tools for the label-free detection of biomolecular interactions and chemical sensing due to their high sensitivity and real-time monitoring capabilities [3,4]. Recent studies have highlighted the diverse applications of SPR sensors in fields such as environmental monitoring [5], medical diagnostics [6,7], and food safety [8]. The development of novel materials and structures to enhance the sensitivity and performance of SPR sensors has become a key research focus in the field of SPR sensing [9,10,11]. The integration of two dimensional (2D) materials, including graphene, transition metal dichalcogenide (TMDC), black phosphorus (BP) and MXene, into SPR sensor platforms has shown great potential for improving sensor performance and expanding their application range [12,13,14,15].

BP, a layered material with a puckered structure, exhibits a tunable bandgap, high carrier mobility, strong light–matter interactions, and exceptional electrical, optical, and mechanical properties, which make it a versatile candidate for various applications, including optoelectronics, photonics, and sensing [16,17,18,19]. The BP hybrid Kretchmann SPR sensors exhibit significant advantages. The BP nanostructures primarily aim to enhance the acceleration of plasmon material signals, resulting in improved molecular sensing. This improvement can be attributed to the 40-times faster-sensing responses of BP nanostructures compared to those reported for other 2D nanomaterials [20]. Wu et al. present the SPR-assisted biochemical sensor with a heterostructure of few-layer BP (FLBP) and graphene/TMDCs, which could achieve the maximum reflectivity sensitivity of 279°/refractive index units (RIU) [21]. Pal et al. proposed a vdW heterostructure using black phosphorus/TMDC/MXene in the SPR sensor for biological detection with high sensitivity of 388°/RIU [22]. Dey et al. proposed a Kretschmann configuration-based heterostructure SPR sensor using WS_2_/metal/WS_2_/graphene by the finite difference time-domain (FDTD) method with the highest sensitivity of 208°/RIU [23]. Zohre et al. proposed a SPR biosensor with a graphene–MoS2 hybrid layer, which achieved a sensitivity of 192°/RIU [24]. Maneesh et al. proposed an angular interrogated anisotropic bimetallic SPR biosensor by stacking anisotropic BP and monolayer antimonene on Cu/Ni bimetallic films with highest sensitivity of 446.90°/RIU [25].

The anisotropic nature of FLBP, arising from its puckered structure and in-plane anisotropy, offers unique opportunities for tailoring the optical properties of SPR sensors [26,27]. Recent research has highlighted the significance of the anisotropic properties of FLBP in enhancing the performance of optical sensors and enabling novel sensing applications [28]. Our previous research already proved that the sensing performance along the two principal crystal axes (zigzag and armchair) of BP show great difference due to the distinct anisotropic nature of black phosphorus [29]. However, most of the current SPR sensors employed the same refractive index parameters along the zigzag (zz) and armchair (ac) principal crystal axes to calculate the sensitivity of heterostructures containing FLBP, while the refractive index of FLBP films along different crystallographic orientations should be distinguished.

This study investigates the impact of different stacking sequences of FLBP on the sensing performance of prism-Ag-FLBP Kretschmann SPR sensors both theoretically and experimentally. In the theoretical aspect, the Fresnel equations are applied to calculate the reflectance, phase, and their sensitivities. Additionally, the transmission matrix method is employed to analyze the electric field distribution at the SPR resonance angle. By modulating the thickness and stacking sequence of FLBP, the highest reflective and phase sensitivitities that could be achieved were 287.9°/RIU and 1162 °/RIU. Specifically, when there are 12 layers, the zz stacking FLBP structure exhibits a reflectivity sensitivity that is 76°/RIU higher than the ac stacking structure. Additionally, the phase sensitivity of the zz stacking FLBP structure is 276.9 °/RIU higher than that of the ac stacking structure. In the experimental aspect, the well-known Attenuated Total Reflection (ATR) θ−2θ SPR setup is utilized to detect the reflectance and phase. Moreover, angle-resolved polarized Raman spectroscopy (ARPRS) is employed to investigate the thickness and crystal orientation of FLBP. Samples of FLBP with the same thickness but different stacking sequences were analyzed. The results revealed significant differences in the resonant angle and sensitivities due to the variation in FLBP stacking sequences. These results highlight the significant impact of anisotropic black phosphorus structures on the sensitivity of the SPR sensors.

## 2. Methods

**Figure 1:**
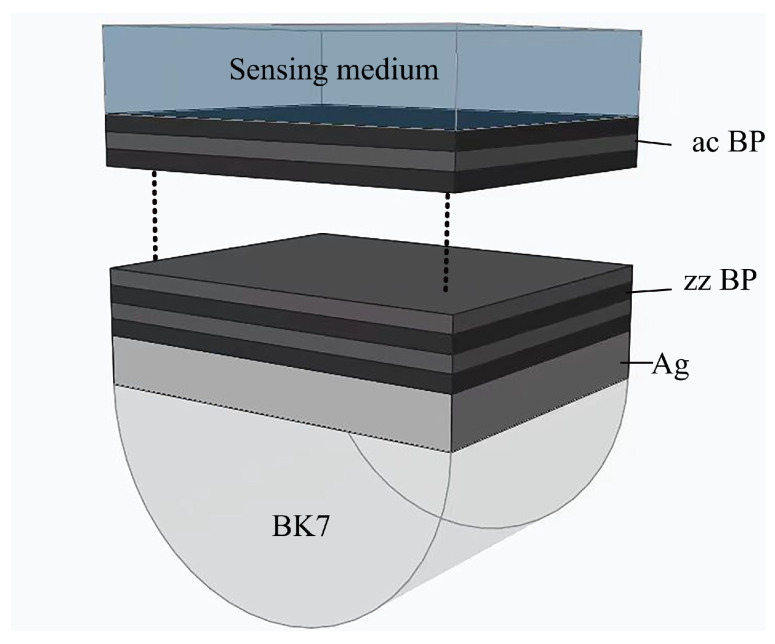
Schematic diagram of BK7-Ag-FLBP SPR sensor.

Figure 1 depicts a schematic of the proposed prism-Ag-FLBP SPR sensors. The wavelength of incident light is 633 nm, which was chosen based on the bandgap of FLBP. This incident light travels through free space and reaches a BK7 prism hemisphere with a refractive index of =1.5151 at λ = 633 nm. A 50 nm thick layer of silver (Ag) is present in the structure [30]. The refractive index of BP in the zigzag (zz) direction is n_zz_ = 3.78389 + 0.10403i, while in the armchair (ac) direction, it is n_ac_ = 3.62584 + 0.40134i [31]. For the sensing medium, the refractive index of 1.33 + Δn is assumed, where Δn is the change of refractive index in the sensing medium due to a biological or chemical reaction.

The phase of p-polarized light and s-polarized light can be calculated by the corresponding reflecttion coefficient:

(1) $$\begin{array}{c} \phi_{p,s}=arg\left(r_{p,s}\right). \end{array}$$

The phase value after eliminating the interference signal:

(2) $$\begin{array}{c} \phi =|\phi_{p}-\phi_{s}|. \end{array}$$

The ratio of the change in the angle of resonance (Δθ) to the change in the refractive index of the sensing medium (Δn) is the reflectivity sensitivity of the sensor, which is defined as:

(3) $$\begin{array}{c} S_{1}=\frac{\Delta \theta }{\Delta n}. \end{array}$$

To maintain stability and repeatability in the sensing process, we define the phase sensitivity as the ratio of the phase difference at the resonant angle ($\Delta \phi_{spr}$) to the change in the refractive index of the sensing medium or analyte (Δn). This is represented as

(4) $$\begin{array}{c} S_{2}=\frac{\Delta \phi_{spr}}{\Delta n}. \end{array}$$

## 3. Experiments

The experimental setup used for measuring phase intensity is the commonly used θ−2θ angular interrogation Kretschmann configurations [32]. In this setup, an excitation He-Ne laser emitting light with a wavelength of 632.8 nm was directed toward a polarizer to ensure an incident p-polarization. The p-polarized light then excites the SPR signal from the silver-FLBP configuration located on the hemispherical BK7 prism. The prism is positioned at the center of the θ−2θ goniometer using a customized holder, ensuring a secure and stable placement. The reflected signal passing through an analyzer was collected by a photodiode. When performing typical angular SPR reflectivity scans, no analyzer is present, while for phase measurements, an analyzer is applied, which is orientated to the s-polarization. We conducted our experiment under ambient conditions of 20 °C, and any temperature fluctuations induced by the laser on the sample surface were not taken into account.

Silver thin films are deposited by vacuum thermal evaporation directly onto the hemi-cylindrical prisms. The FLBP was obtained by mechanical exfoliation by using the adhesive tape to peel off thin flakes of BP from the crystal’s surface and then transfer the flakes onto the Ag pre-coated prism. FLBP samples were obtained from XFNANO Materials Tech Co., Ltd. (Nanjing, China). For the sake of convenience as a control, open air was employed as the dielectric analyte.

Angular-resolved polarized Raman spectroscopy (ARPRS) at room temperature was applied to identify the crystalline orientation of two different FLBP samples, which have already been pre-coated to the prism-Ag structure. The system is in a backscattering configuration. A 532 nm incident laser light, which is additionally filtered against amplified spontaneous emission (ASE), shines onto a rotational sample stage with an objective lens to collect scattered signal. A rotatable polarizer is applied for polarization analysis before an extreme low-frequency filter. Finally, the collimated light is dispersed by a high-throughput f/1.8 spectrometer and mapped onto a deep cooled EMCCD camera. Detailed FLBP thickness and stacking sequences assignments could be found in our previous work [33].

## 4. Results

### 4.1. Theoretical Analysis

**Figure 2:**
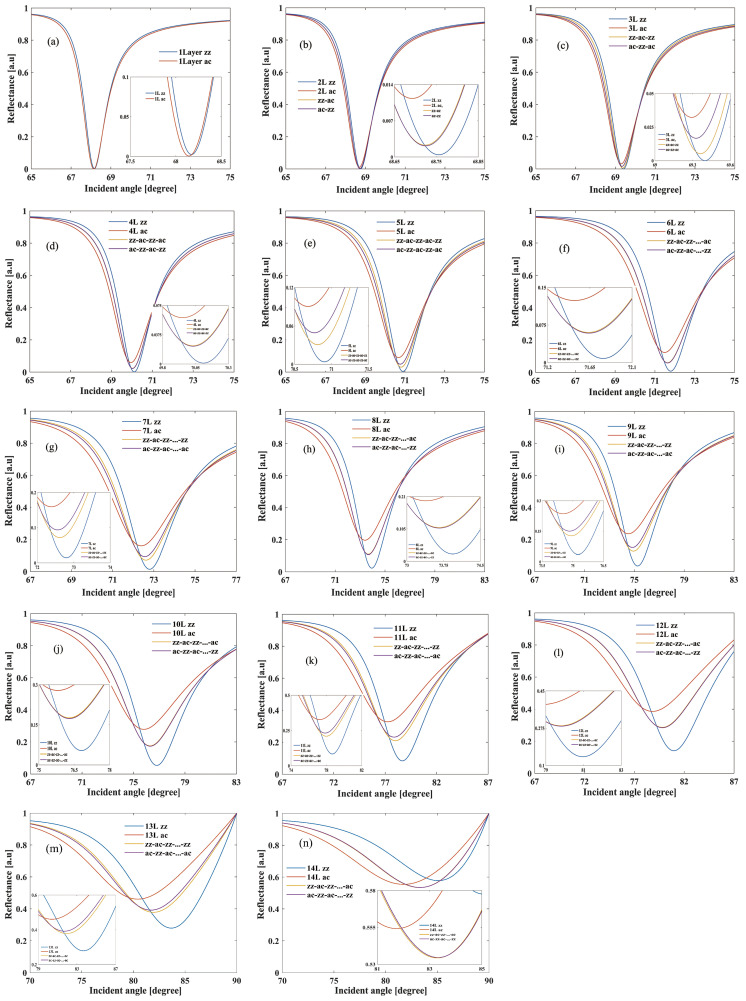
Reflectance curves of the BK7-Ag-FLBP SPR structure for different stacking sequences (zz, ac, zz-ac, and ac-zz) comprising 1 to 14 layers of FLBP with a refractive index of 1.33 for the sensing medium. (**a**–**n**) represent the layer numbers of FLBP, ranging from 1 layer to 14 layers.

We conducted a systematic exploration of both SPR reflectivity and phase in the suggested BK7-Ag-FLBP structure by varying the stacking sequence and the number of FLBP layers. Figure 2 illustrates the reflectivity of our SPR structure based on four types of stacking configurations (zz, ac, zz-ac, and ac-zz) of 1 to 14 layers of FLBP. The reflectivity results near the SPR resonant angle have been amplified, as depicted in the illustration of each figure. Clear resonant angle shifts caused by different stacking sequences of FLBP can be found. Specifically, regardless of the variation in the number of stacked layers, the reflectance of the zz-stacked structure consistently exhibits lower reflectance at the resonance angle, while the ac-stacked structure always presents higher reflectance. It is worth noting that under even-numbered layer stacking, the reflectivity curves of zz-ac-zz and ac-zz-ac stacking configurations almost overlap. Under an odd-numbered layer stacking, the reflectivity curves of zz-ac-zz and ac-zz-ac stacking configurations exhibit dispersion near the resonance angle. Apart from the stacking sequences, the stacking layer number also affects the resonant angle. As the number of BP layers increased, the incident angles exhibited a significant red shift for a fixed Ag film thickness (50 nm), resulting in broader SPR curves due to electron energy loss from the deposition of BP layers. Therefore, the minimum reflectivity was strongly linked to the number of BP layers and the stacking sequences on the Ag film.

**Figure 3:**
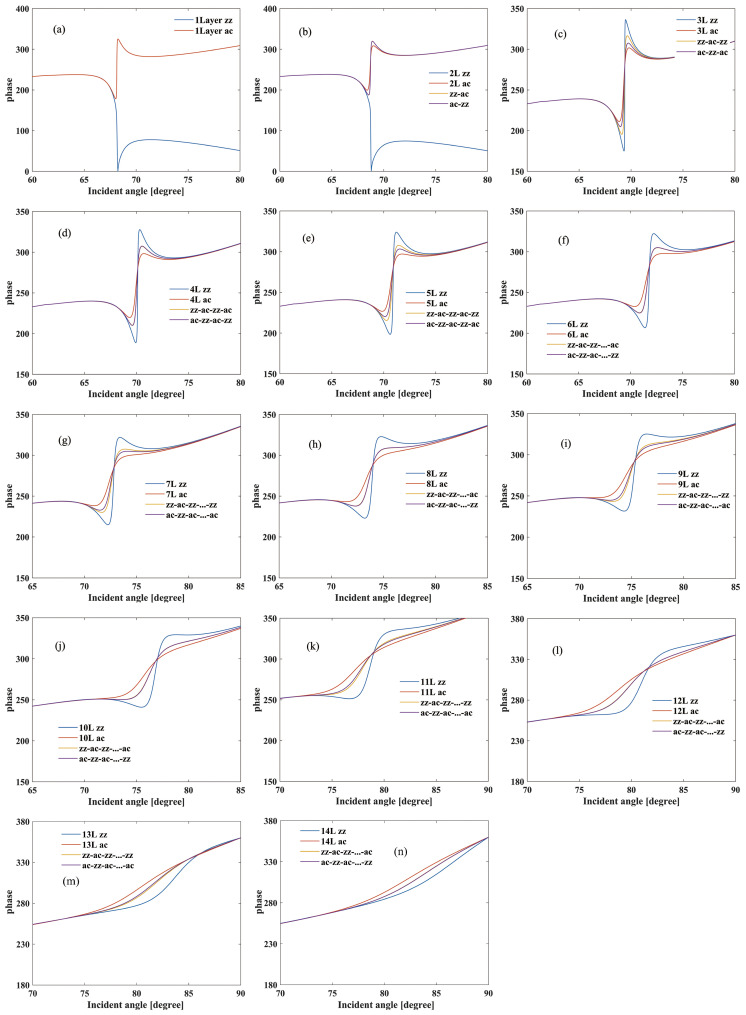
Phase curves of the BK7-Ag-FLBP SPR structure for different stacking sequences (zz, ac, zz-ac, and ac-zz) comprising 1 to 14 layers of FLBP and a refractive index of 1.33 for the sensing medium. (**a**–**n**) represent the layer numbers of FLBP, ranging from 1 layer to 14 layers.

In addition to the variations in reflectivity, there is a conspicuous phase shift observed in the reflected light at the SPR resonant angle, as shown in Figure 3. Similar to the reflectivity, structures with zz stacking exhibit a more significant phase shift, whereas those with ac stacking display a less phase shift. As the layer number increases, the phase shift at SPR resonant angles decreases. When the number of layers is even, the phase variations based on zz-ac and ac-zz stacking sequences are quite similar. However, when the number of layers is odd, the phase variation curves no longer overlap. This is due to there being a significant charge transfer between FLBP and the Ag film, which is strongly dependent on the number of BP layers. Increasing the number of layers leads to a clear decreasing trend in carrier effective masses, indicating higher mobility for FLBP with larger layer numbers compared to smaller layer numbers. Moreover, the hole mass exhibits a notable dependence on thickness along the zig-zag direction due to interlayer coupling [28,34].

**Figure 4:**
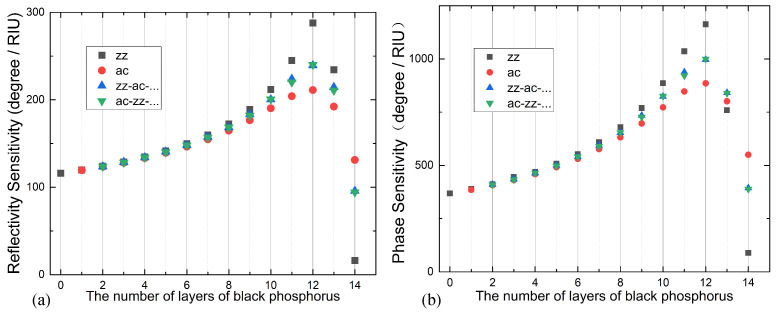
(**a**) Reflectivity sensitivity and (**b**) phase sensitivity of the BK7-Ag-FLBP SPR sensor for different FLBP stacking sequences ranging from 0 to 14 layers. The stacking sequences are represented as follows: black squares for zz stacking, red dots for ac stacking, blue upright triangles for zz-ac stacking, and green inverted triangles for ac-zz stacking.

The reflectivity sensitivity variation due to the stacking sequence and thickness has been analyzed in detail, shown in Figure 4a. Overall, the reflectivity sensitivity of our structure varies depending on the number of stacking layers. It increases as the layer number increase up to 12 layers, reaching its peak sensitivity of 287.9°/RIU. However, beyond 12 layers, the sensitivity rapidly decreases. Additionally, when the number of stacking layers is relatively low, such as less than eight layers, the sensitivity exhibits a nearly linear relationship with the layer number irrespective of the stacking sequence. However, with more than eight layers, the sensitivity is significantly influenced by the stacking sequence. The zz stacking structure exhibits the highest sensitivity, followed by the zz-ac cross-stacking structure, while the ac stacking structure has the lowest sensitivity. Notably, when there are 12 layers, the zz stacking structure shows a sensitivity that is 76°/RIU higher than the ac stacking structure, emphasizing the impact of anisotropic black phosphorus structures on sensor sensitivity.

When the refractive index of the sensing medium changes from 1.33 to 1.335 (Δn = 0.005), the variation pattern of phase sensitivity is similar to that of angular sensitivity modulation, as shown in Figure 4b. As the layer number increases, the phase sensitivity also increases and reaches its maximum at 12 layers. However, if the number of layers continues to increase, the sensitivity will rapidly decrease. In the case of a 12-layer BP SPR sensor in the zz direction, the phase sensitivity reaches its maximum at 1162°/RIU, which is 3.16 times higher than that of traditional Ag-based SPR sensors. However, in the ac direction of the same structure, the phase sensitivity is much lower with a difference of 276.9°/RIU.

The sensitivity and resonance angle of SPR sensors with the same structure vary significantly with different sensing media. Determining the stacking sequences of BP films can be a cumbersome process. In conventional coating techniques, there is a high degree of randomness in the twisting angles between different layers of FLBP. For instance, in the case of a 12-layer ac structure used for detecting a sensing medium with a refractive index of $n_{s}=1.33$, the SPR angle is approximately 78.825°. However, if we assume the structure to have a zz-stacking arrangement, the detected refractive index of the sensing medium becomes 1.324, which indicates a completely different type of liquid. This example clearly demonstrates the crucial importance of comprehending the anisotropic properties of FLBP-based SPR sensors.

The aforementioned analysis of reflectivity, phase shift, and their sensitivities reveals that the anisotropic structure of FLBP plays a significant role in influencing the sensing performance of BP-SPR sensors. This is because when FLBP comes into contact with the Ag film, charge transfer takes place between FLBP and the Au film, ensuring the continuity of their Fermi levels. The intensity of this charge transfer is highly dependent on the number and stacking sequences of BP layers. To enhance the sensitivity of an SPR sensor, a strong charge transfer between FLBP and the Ag film is required. Therefore, understanding and controlling the stacking number and sequences of FLBP are essential for optimizing the sensing performance of FLBP-based SPR sensors. Table 1 presented below provides an overview of three previously reported surface plasmon resonance (SPR) sensors based on BP. However, it is worth noting that previous studies only focused on the thickness of the BP layer and did not consider the stacking sequences, which actually exhibit significant differences in sensitivity as demonstrated in our work. Clearly, the BK7-Ag-12-layer zz stacking BP structure in our study shows higher reflectivity sensitivity compared to these previous works.

The intensity of reflected light is greatly reduced as the incident light energy is predominantly absorbed by the surface plasmon wave. Alongside the charge-transfer effect facilitated by the BP layers, a considerably amplified electric field is present at the sensing interface of silver–BP. This enhanced electric field phenomenon is also observed in other isotropic 2D materials, including graphene and TMDCs, which have been utilized to enhance sensing substrates.

When SPR occurs, the energy of the electric field is mainly distributed near the interface of the sensing medium layer. The sensitivity of the SPR structure is related to electric field strength, but it is also necessary to consider the light absorption ability of 2D materials. Among the SPR sensor structures we discussed, the angular sensitivity of 12-layer BP is the highest. Under the condition of 12-layer BP, there are also significant differences among the samples with four different stacking sequences. Normalize the incident electric field to obtain the electric field distribution at the corresponding resonance angles for the four stacking methods of the Ag-12-layer BP sensor.

**Figure 5:**
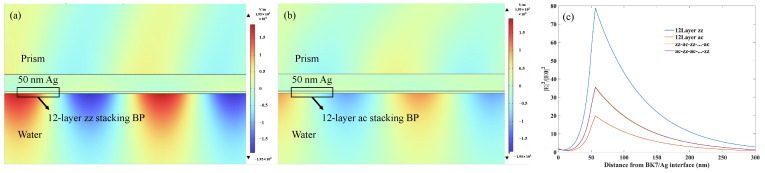
The electric field distribution of sensing substrate composed of 12-layer BP with (**a**) zz stacking and (**b**) ac stacking, and 50 nm Ag film under the resonance condition. (**c**) Normalized electric field at the corresponding resonance angles for the four stacking methods of the Ag-12-layer BP sensor.

As shown in Figure 5a,b, the excited SPR electric field of the 12-layer zz stacking BP structure is much higher than that of the ac stacking structure. Figure 5c illustrates the normalized electric field at the corresponding resonance angles for the four stacking methods of the Ag-12-layer BP sensor. The maximum electric field intensity of different structures is directly proportional to the sensitivity of the corresponding structures. The sensitivity of the sensors for the other two cross-stacked BP structures is similar, and their corresponding highest electric field intensity and electric field distribution at resonance are also very similar. This further indicates that a stronger electric field can make the evanescent wave more sensitive and detect changes in the refractive index of the sensing medium. From the concept of penetration depth, it can be inferred that the larger the maximum electric field intensity, the wider the range of the sensing medium being detected. Therefore, in the structural design, to improve sensitivity, we need to consider to some extent the distribution of the electric field when the sensor experiences resonance.

### 4.2. Experimental Analysis

**Figure 6:**
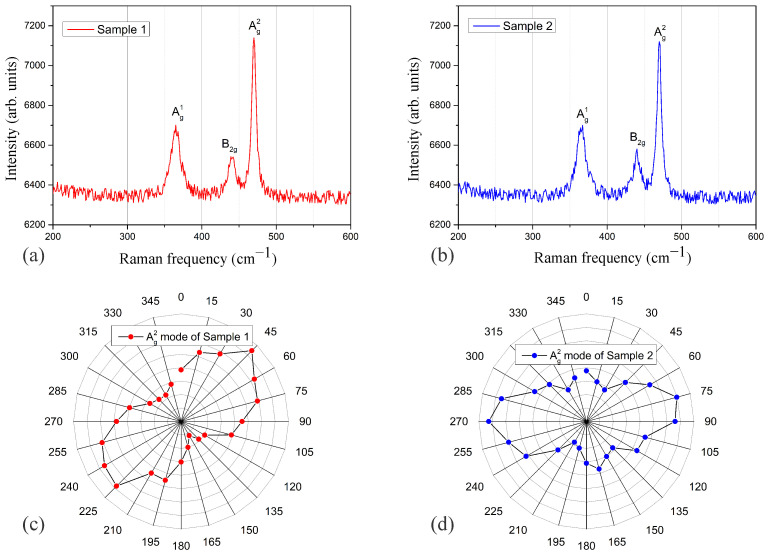
(**a**,**b**) represent Raman spectroscopy of FLBP coated on the Ag pre-coated prism of two different samples. (**c**,**d**) show the polarization analysis of the $A_{g}^{1}$ mode of the two samples, where (**c**) corresponds to the polarization analysis of (**a**), and (**d**) corresponds to the polarization analysis of (**b**).

Figure 6a,b depict the Raman spectroscopy results of two different FLBP samples. It can be observed that both samples exhibit distinct peaks $A_{g}^{1}$ (365 cm^−1^), $B_{2}g$ (441 cm^−1^), and $A_{g}^{2}$ (470 cm^−1^). The Raman frequencies and intensities of the three peaks in both FLBP samples are nearly identical, suggesting that the thickness of the two FLBP samples is almost the same. However, upon adding a polarizer to the scattering signal path, the two samples display completely different polarization dependencies, as shown in Figure 6c,d. This discrepancy indicates different crystalline orientations or stacking orders between the two samples. Based on this observation, we proceeded with SPR reflectivity and phase measurements using these two samples for comparison.

**Figure 7:**
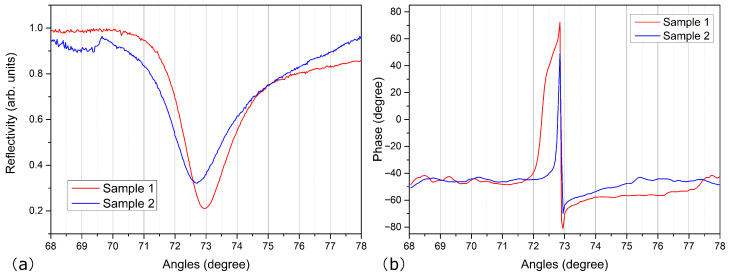
Experiment measurement of (**a**) the reflectivity and (**b**) phase of the prism-Ag-FLBP structure for the two different FLBP samples.

Figure 7 illustrates the experiment measurement of the reflectivity and phase of the prism-Ag-FLBP structure with the above two FLBP samples. Clearly, the structure with sample 1 exhibits a lower SPR dip and a larger phase shift compared to sample 2, indicating that sample 1 can induce a stronger SPR effect. The SPR resonant angle difference between the two samples is approximately 0.275°, while the maximum phase difference shows a difference of approximately 34.6°. Although the crystal orientations of the two samples are not exactly in accordance with the ac or zz stacking as analyzed in the theoretical calculation, the ARPRS analysis has confirmed that the two samples possess completely different stacking sequences. Therefore, these experimental results provide evidence that the stacking sequences of FLBP have a significant impact on the sensing performance of SPR sensors. In view of this, despite the difference in analytes employed in the experiment and calculation, both analyses demonstrate that SPR sensors with the same thickness of FLBP but different stacking conditions show significant variations in reflectivity and phase.

## 5. Conclusions

In conclusion, our systematic exploration of the BK7-Ag-FLBP structure for SPR sensors has provided valuable insights into the influence of stacking sequences and layer numbers on the reflectivity and phase characteristics. The theoretical analysis revealed significant differences in reflectivity and phase sensitivity between the zz and ac stacking sequences with maximum values of 76°/RIU and 276.9°/RIU, respectively. In the experimental analysis, a resonant angle difference of approximately 0.275° was observed for FLBP samples with the same thickness but different stacking sequences.

This discrepancy highlights the impact of the anisotropic properties of FLBP on the accuracy of SPR sensors. For future work, precise control over the stacking sequences of FLBP in the experiment would be beneficial for achieving higher sensing performance and minimizing inaccuracies caused by anisotropy. Overall, understanding and controlling the stacking thickness and sequences are essential for optimizing the design and performance of SPR sensors based on FLBP-like anisotropic materials, paving the way for innovative applications in various fields.

## Figures and Tables

**Table 1:** Comparison with the earlier reported work.

Sensor Structure	Number of BP Layers	Reflectivity Sensitivity (°/RIU)	References
BK7-Au-Mxene-WS2-BP	1	190.22	[35]
BK7-Ag-BP-MXene	6	264	[36]
BK7- Ag-BP-WSe2	9	279	[21]
BK7-Ag-FLBP	12 (zz stacking)	287.9	this work
	12 (ac stacking)	211.1	this work
	12 (ac-zz stacking)	240.1	this work
	12 (zz-ac stacking)	239.3	this work

## Data Availability

The data presented in this study are available on request from the corresponding author upon reasonable request.
